# Importance of allergic sensitisation for normal range of fractional exhaled nitric oxide in adolescents

**DOI:** 10.1111/pai.70154

**Published:** 2025-08-01

**Authors:** Jenny Hallberg, Gang Wang, Anna Bergström, Inger Kull, Erik Melén, Andrei Malinovschi

**Affiliations:** ^1^ Sachs' Children and Youth Hospital, Södersjukhuset Stockholm Sweden; ^2^ Department of Clinical Science and Education Södersjukhuset and Karolinska Institutet Stockholm Sweden; ^3^ Division of Internal Medicine, Institute of Integrated Traditional Chinese and Western Medicine, West China Hospital Sichuan University Sichuan China; ^4^ Institute of Environmental Medicine Karolinska Institutet Stockholm Sweden; ^5^ Department of Medical Sciences, Clinical Physiology Uppsala University Uppsala Sweden

**Keywords:** asthma, epidemiology, IgE sensitisation, nitric oxide, pulmonary function tests

## Abstract

**Background:**

In addition to body anthropometrics, allergen sensitization has been shown to influence the levels of exhaled fractional nitric oxide (FeNO) in adults, but whether this relationship is valid also in younger age groups, how it compares to the fixed cut‐off levels recommended for clinical practice, and relates to respiratory symptoms remains to be confirmed.

**Method:**

FeNO, sensitization, respiratory symptoms, and spirometry were evaluated in 2054 adolescents taking part in the 16‐year follow‐up of the Swedish BAMSE birth cohort.

**Results:**

The median and upper limit of FeNO was related to the presence and type of allergic sensitisation and poorly represented by one fixed cutoff. For example, the 95th percentiles for sensitisation to dogs were 89 ppb in females and 87 ppb in males. A FeNO value above the sensitisation‐specific 95th percentile was, in those with allergic sensitisation, related to significantly higher odds for current asthma (OR 4.10, 95% CI 2.44–6.89) and wheeze (OR 2.58, 95% CI 1.44–4.62). In those without allergic sensitisation, a FeNO above the 95th percentile was related to wheeze (OR 3.20, 95% CI 1.37–7.51). The odds for having airway obstruction were not related to any of the FeNO cutoffs for either of the groups.

**Conclusion:**

We have shown that the FeNO upper limit of normal is dependent on the presence and type of allergic sensitization and that these cutoffs differ from the fixed cutoffs of 25 and 50 ppb recommended in the literature. Sensitization specific rather than fixed limit cutoffs may be more relevant to asthma diagnosis and asthma symptoms in a general adolescent population.

**Key message**

Our study provides insights in the effect of degree and type of allergic sensitization on the fractional exhaled nitric oxide upper limit of normal. Our work provides evidence for individualized fractional exhaled nitric oxide cutoffs with regard to allergic sensitization. These individualized cutoffs might be more relevant in the work‐up of asthma diagnosis, and this needs to be further studied.

## 1 BACKGROUND

Fractional exhaled nitric oxide (FeNO) is a biomarker of type‐2 airway inflammation, reflecting activation of respiratory epithelial inducible nitric oxide synthetase, and is increasingly used in clinical practice for diagnosis and management of asthma.^1^, ^2^, ^3^ Current clinical practice guidelines from the American Thoracic Society state that values between 25 and 50 ppb should be interpreted with caution in adults and children >12 years, and values above a fixed cut‐off limit of 50 ppb indicate eosinophilic and steroid‐responsive inflammation.^4^ This last named cut‐off is also used by the recent European Respiratory Society (ERS) guidelines for asthma diagnosis in adults^2^ and for both biomarker‐supported asthma diagnosis for persons aged >16 years in the recent guidelines from British Thoracic Society/National Institute for Health and Care Excellence /Scottish Intercollegiate Guidelines Network.^5^ The ERS guidelines for diagnosis of asthma in children suggest a different use of FeNO in order to reinforce asthma suspicion if levels above 25 ppb are found and therefore continue the diagnostic workup of asthma.^1^

However, FeNO has been shown to be influenced by a number of factors, such as age, sex, and height,^6^ explaining around 12% of the FeNO variation, as recently reported by a GLI ERS task force.^7^ Another factor known to be related to elevated FeNO levels is allergic sensitisation, where the size of the effect varies across studies from 15% to 60%.^8^ In 50‐ to 64‐year‐old adults in the Swedish CArdioPulmonary bioImage Study (SCAPIS), both the degree and type of allergic sensitisation were found to significantly affect the upper limit of normal (ULN) of FeNO.^9^ Furthermore, IgE sensitisation to perennial allergens and a higher degree of IgE sensitisation (defined as higher IgE antibody levels to a mix of aeroallergens) resulted in higher median and ULN of FeNO compared with non‐atopic subjects, indicating that the degree and type of IgE sensitisation are important to consider in the generation of reference values of FeNO in adults. Whether this is relevant also in younger age groups remains to be studied.

Therefore, the aim of the current study is to extend the knowledge and study how allergic sensitization affects the median and ULN for FeNO in adolescents, and how different cutoffs are related to the prevalence of asthma symptoms and lung function impairment in this younger age group.

## 2 METHOD

### 2.1 Study population

The BAMSE (Swedish abbreviation for Children, Allergy, Milieu, Stockholm, Epidemiology) study is a prospective birth cohort including 4089 Swedish children.^10^ Parents of all infants born between 1994 and 1996 in predefined areas of Stockholm, including inner city, urban, and suburban districts, were asked to participate in the study. The original cohort consists of 75% of eligible children. Baseline characteristics were collected by parental questionnaires when the children were approximately 2 months of age (time of inclusion). Parental reports on respiratory symptoms and medication were obtained by questionnaires answered at ages 1, 2, 4, 8, and 16 years. In addition, smoking habits and symptom severity were reported by the adolescents at 16 years. Response rates ranged from 96% to 78% at each occasion. At 16 years of age, all subjects were invited to attend a clinical follow‐up.

### 2.2 Reference population

The reference population consisted of non‐smoking individuals without current wheeze (in the last 12 months), a history of physician‐diagnosed asthma, or any asthma medication (in the last 12 months) reported in the questionnaire at 16 years of age.

### 2.3 Clinical examination

The clinical examination at 16 years included anthropometry, blood sampling, measurements of FeNO, and spirometry.

FeNO was measured with a chemiluminescence analyzer (EcoMedics Exhalyzer® CLD 88sp with Denox 88, Eco Medics, Duernten, Switzerland). The procedure was performed in accordance with published guidelines.^11^ Mean exhalation flow rate was 50 mL/s ± 10% during the NO plateau. The mean value of two measurements within 10% of the mean was used for analysis. FeNO was defined as the mean of these values expressed in ppb. The analyzer was calibrated using a standard NO calibration gas (Air Liquide Deutschland GmbH, Krefeld, Germany).

Spirometry was performed using the Jaeger MasterScreen system (Carefusion Technologies, San Diego, California). All subjects performed more than one maximal expiratory flow volume (MEFV) measurement. The highest values of forced vital capacity (FVC) and forced expiratory volume in 1 s (FEV_1_) were extracted and used for analysis, provided that the subject's effort was accepted as being maximal by the test leader, the MEFV curve passed visual quality inspection, and that the two highest FEV_1_ and two highest FVC readings were reproducible according to ATS/ERS criteria.^12^ Values were then converted to *z*‐scores using the reference equations by Quanjer et al.^13^

### 2.4 Blood analyses

Sera were analyzed for immunoglobulin E (IgE) reactivity to a mix of common inhalant allergens (timothy, birch, cat, dog, house dust mites *(Dermatophagoides pteronyssinus and/or Dermatophagoides farina)*, mugwort, horse, and *Cladosporium herbarum*) using Phadiatop® (ThermoFisher Scientific/Phadia AB, Uppsala, Sweden; Department of Clinical Immunology, Karolinska University Hospital, Stockholm).^14^ *Allergic sensitization* was defined as an IgE level of ≥0.35 Phadia Arbitrary Units (PAU)/liter (L) to Phadiatop®. In allergic sensitized (Phadiatop positive) individuals, specific sensitization was further tested individually toward abovenamed allergens included in the Phadiatop (ThermoFisher Scientific/Phadia AB, Uppsala, Sweden) and reported as positive if the IgE level was ≥0.35 kilounits (kU)/L.^15^

### 2.5 Variable definitions

#### 2.5.1 Smoking status

Smoking status was obtained from the adolescent's own questionnaire answers. Any smoking (daily or occasionally) was classified as active smoking.

*Asthma* was defined according to the Mechanisms of the Development of ALLergy (MeDALL) definition as a positive answer to at least two of the following: ever had doctor‐diagnosed asthma, wheezing within the last 12 months, and use of any asthma medication within the last 12 months.^16^

### 2.6 Statistical analysis

Comparisons between groups regarding background variables, FeNO, and prevalence of specific symptoms were performed by the chi‐squared test. All further analyses were performed in non‐smoking subjects. The relationship between FeNO and age, height, and weight was assessed for the 50th (median) and 95th (ULN) percentiles in the reference population using quantile regression, stratified by sex. Significant anthropometric variables were then adjusted for in the model analyzing the influence of specific sensitization patterns on FeNO levels. The odds of asthma symptoms and lung function impairment were calculated using odds ratios. *p*‐values of <.05 were considered statistically significant. Analyses were performed using the Stata 15.1 software package (StataCorp LP, College Station, TX, USA).

The BAMSE study was approved by the Regional Ethical Review Board in Stockholm, and all parents and participants provided consent to participate in the study.

## 3 RESULTS

**FIGURE 1:**
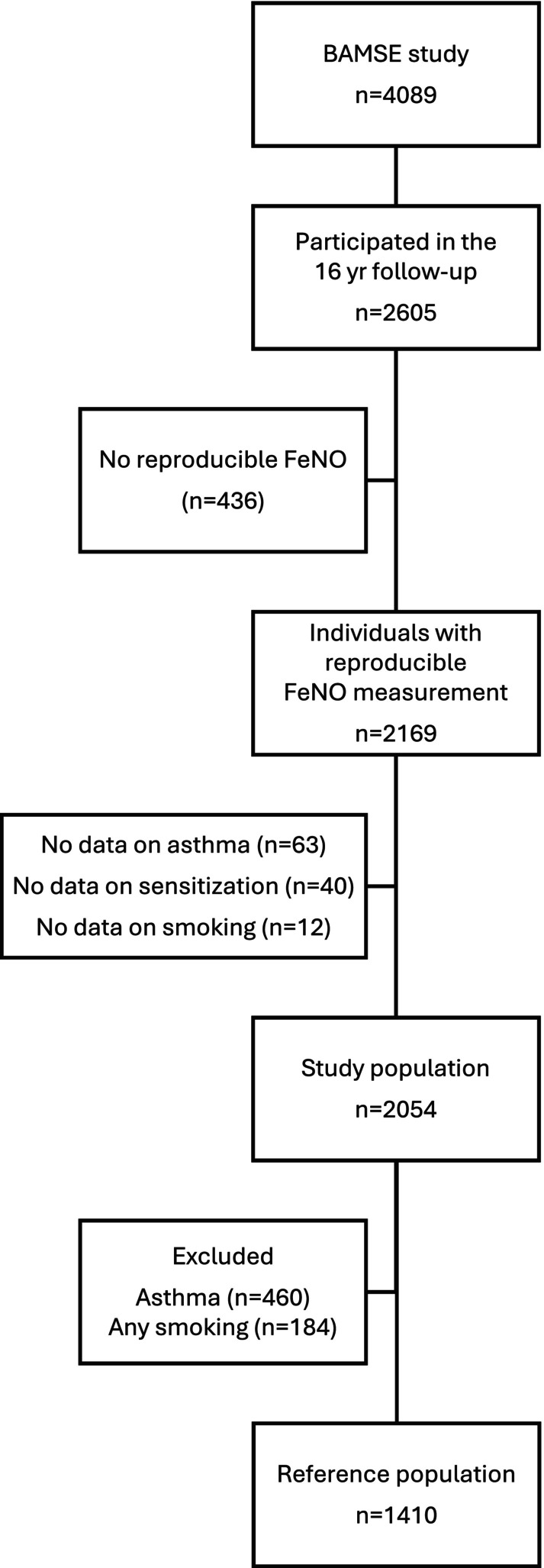
Flowchart of the BAMSE population relevant for the present study.

A total of 2605 BAMSE participants (64% percent of the original cohort) took part in the clinical examination at 16 years of age (Figure 1). General baseline characteristics for the study population with FeNO data, information on smoking habits, respiratory symptoms, and sensitization available (*n* = 2054), as well as the non‐symptomatic, non‐smoking reference population (*n* = 1410) are presented in Table 1. A total of 39 among the 143 subjects with asthma (eg 27.3%) with available lung function data had obstructive spirometry. Among Phadiatop positive subjects with asthma, the proportion was higher (27 of 64, eg 42.2%).

**TABLE 1:** Characteristics at the 16 years' follow‐up.

	Study population (*n* = 2054)	Reference population (*n* = 1410)
Age (years)	16.7 (0.4)	16.7 (0.4)
Sex (% male)	48.7	48.8
Height (cm)	173.4 (9.0)	173.5 (9.1)
Weight (kg)	65.4 (11.6)	65.0 (11.4)
BMI (kg/m^2^)	21.7 (3.5)	21.5 (3.0)
Active smokers (%)	11.4	0
IgE sensitized^a^ (%)	43.3	38.2
Current asthma (%)	13.0	0

*Note*: Values are presented as mean(±SD) or percentages.

^a^ Sensitisation defined as IgE levels against a mix of aeroallergens (Phadiatop) ≥0.35 Phadia arbitrary units (PAU)/L.

In the reference population, the 50th percentile FeNO values were significantly associated with age and height in females, and weight in males, while no significant associations were found between anthropometric values and the 95th percentiles of FeNO in either sex (Table S1).

**FIGURE 2:**
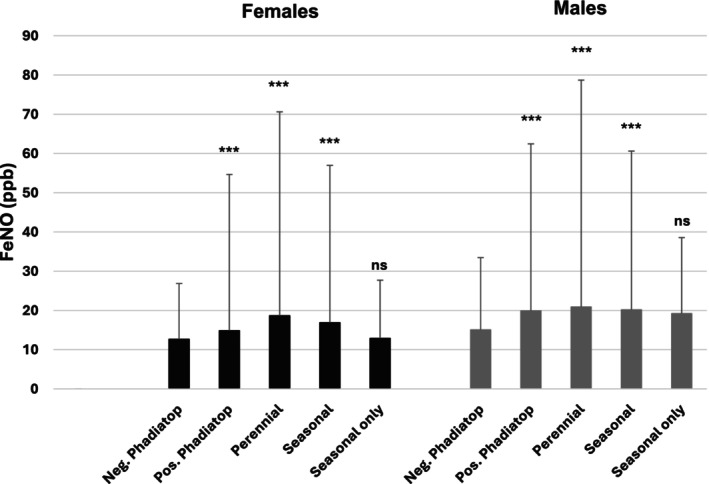
FeNO (ppb) according to type of allergen sensitization in males and females separately, compared to the negative Phadiatop group. *** *p* < 0.001.

The 50th and 95th percentiles of FeNO are shown for males and females by sensitization status in Figure 2 and Table S2. The 95th percentiles of FeNO for non‐sensitized females and males were 27 and 33 ppb, respectively, with sensitized individuals presenting with significantly higher values (males 55 and females 62 ppb). When assessed by allergen, sensitization to furry animals (95th percentiles for females: dog 89 ppb, cat 71 ppb and horse 126 ppb; males: dog 87 ppb, cat 90 ppb, and horse 101 ppb, all *p* < .001) and house dust mites (95th percentiles 75 and 85 ppb for females and males, respectively) were associated with higher FeNO values compared to the non‐sensitized group. The effect of sensitization to seasonal allergens was largely driven by that to perennial allergens (95th percentiles for seasonal allergens: 57 and 61 ppb for females and males respectively, both *p* < .001; after exclusion of those with perennial sensitisation: 28 and 39 ppb for females and males respectively, both *p* = n.s.).

**FIGURE 3:**
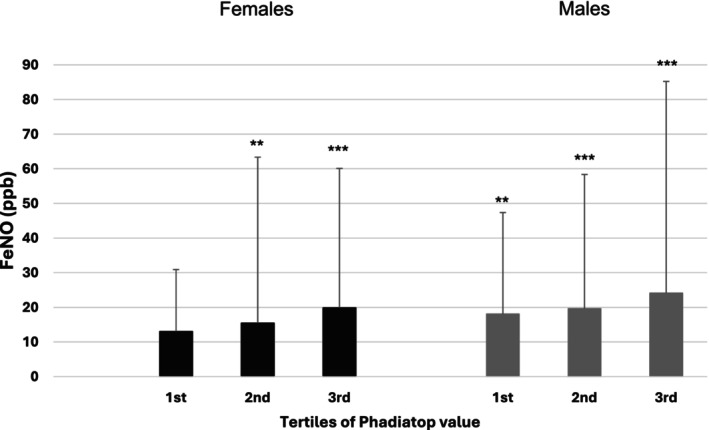
FeNO (ppb) according to degree of allergen sensitization by sex (females 1st tertile <0.85 PAU/L, 2nd tertile 0.85–9.28, 3rd tertile >9.28 and males 1st tertile <2.35, 2nd tertile 2.35–16.7, 3rd tertile >16.7), compared to the negative sensitization group. ** *p* < 0.01, but *p* ≥ 0.001. *** *p* < 0.001.

The degree of IgE sensitization was also associated with the 50th and 95th percentile FeNO values from the first tertile in males (95th percentiles: 1st tertile 47 ppb, *p* = .003; 2nd 58 ppb, *p* < .001; 3rd 85 ppb, *p* < .001) and the second tertile in females (95th percentiles: 1st tertile 31 ppb, *p* = n.s.; 2nd 63 ppb *p* = .001; 3rd 60 ppb, *p* < .001), when compared to the non‐sensitized individuals (Figure 3, Table S3).

**FIGURE 4:**
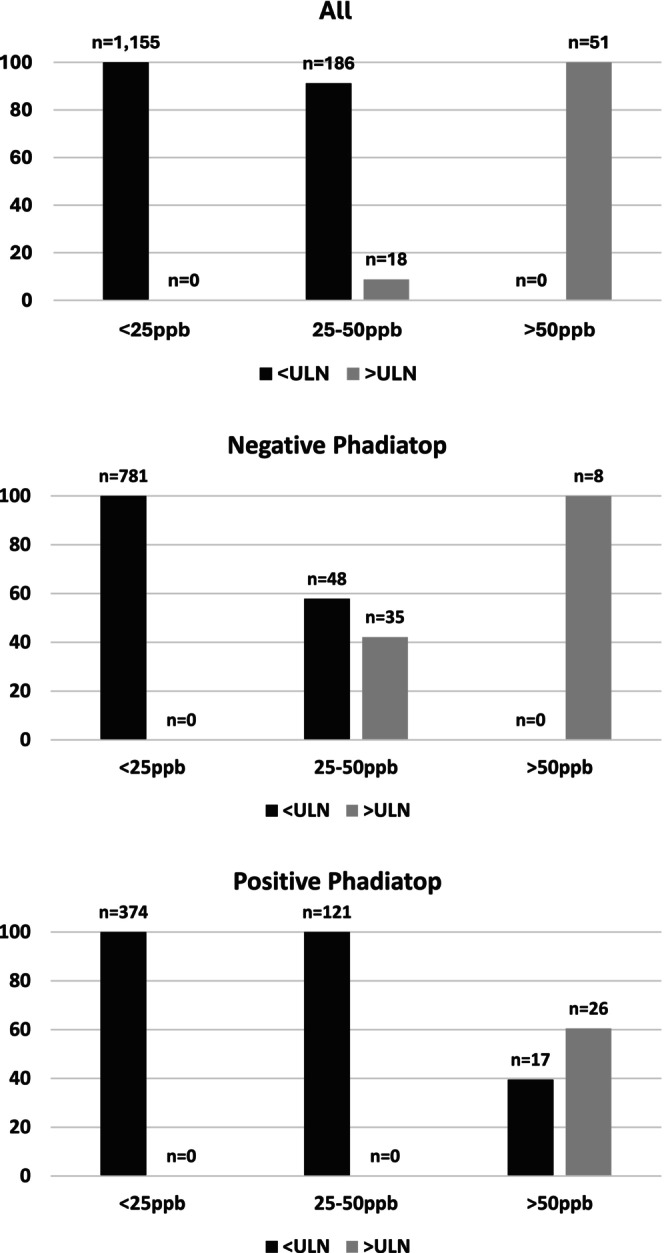
Proportion of individuals with FeNO below and above the upper limit of normal at the 95th percentile calculated for the specific subgroup, and classified as having low, intermediate, or high FeNO according to guidelines, by sensitization status.

The proportions of individuals with FeNO above and below the 95th percentile in groups of low FeNO (<25 ppb), intermediate FeNO (25–50 ppb) and high FeNO (>50 ppb) is further described by sensitisation status in Figure 4. For the non‐sensitized group, the intermediate group consisted to 58% of individuals with a FeNO <95th percentile. Only 8 out of a total of 43 individuals with FeNO over the 95th percentile qualified as “high FeNO.” In contrast, in the sensitized group, 40% of individuals in the high FeNO group had a value below the 95th percentile.

Using the full study population, the probability of concurrent asthma symptoms and airway obstruction was assessed by sensitization status and FeNO cutoff (Table 2). Having elevated FeNO (by all used cutoffs) related to a higher likelihood of having reported wheeze, both among non‐sensitized and sensitized individuals (Table 2). Similar associations were found when looking at reporting at least three episodes of wheezing during the last year: FeNO >25 ppb OR 2.54 (0.99, 6.51) and FeNO >95th percentile OR 5.80 (2.22, 15.14) among non‐sensitized individuals while the corresponding numbers were OR 1.95 (1.19, 3.18) for FeNO >25 ppb, OR 3.08 (1.73, 5.48) for FeNO >50 ppb, and OR 2.62 (1.33, 5.18) for FeNO >95th percentile among sensitized individuals. Having elevated FeNO, using any of the three cutoffs (25, 50, and 95th percentile) was related to having current asthma (Table 2). On the other hand, having elevated FeNO was not significantly related to current asthma in non‐sensitized individuals. The odds for having airway obstruction were not related to having a value below or above either FeNO cutoff for any of the groups (Table 2).

**TABLE 2:** Prevalence and odds ratios with 95% confidence interval for reporting wheeze in the last 12 months, current asthma, or having impaired lung function at 16 years in relation to a FeNO value below or above the sex and sensitization status‐specific 95th percentile, or the 25 ppb cutoff, or 50 ppb cutoff, stratified by sensitization status.

	Wheeze	Current asthma	Airway obstruction^a^
Not IgE‐sensitized (negative to Phadiatop)			
Prevalence	47/1012 (4.64%)	67/1012 (6.62%)	63/734 (7.90%)
FeNO >25 ppb	**2.21 (1.07–4.58)**	1.22 (0.59–2.55)	1.52 (0.75–3.11)
FeNO >27/34 ppb^b^	**3.20 (1.37–7.51)**	2.08 (0.91–4.80)	0.82 (0.25–2.73)
FeNO >50 ppb	n/a^c^	n/a^c^	n/a^c^
IgE‐sensitized (positive to Phadiatop)			
Prevalence	114/807 (14.13%)	174/807 (22.56%)	59/638 (9.25%)
FeNO >25 ppb	**2.02 (1.35–3.02)**	**1.84 (1.31–2.59)**	0.79 (0.44–1.41)
FeNO >50 ppb	**2.76 (1.67–4.54)**	**3.52 (2.26–5.48)**	1.23 (0.58–2.60)
FeNO >55/63 ppb^b^	**2.58 (1.44–4.62)**	**4.10 (2.44–6.89)**	0.94 (0.36–2.45)

*Note*: Bold values indicates *p* < 0.05.

^a^ Airway obstruction defined as FEV_1_/FVC *z*‐score below LLN (−1.64 *z*‐score), using the reference values from Quanjer et al. 2012. Data were available for 1633 individuals.

^b^ Phadiatop negative: 95th percentile in females: 26.85 ppb, males: 33.45 ppb, Phadiatop positive: 95th percentile in females: 54.65 ppb, males: 62.45 ppb.

^c^ Calculations for OR not performed as less than 4 individuals had a FeNO value over the defined limit.

## 4 DISCUSSION

We have shown that, in a general population of adolescents, the median and 95th percentile of FeNO were highly dependent on type and level of IgE sensitisation to inhalant allergens. The highest values were seen in those with sensitisation to furry pets, assessed by specific sensitisation to cat and dog. Furthermore, accounting for sensitisation‐specific 95th percentile instead of fixed cutoffs leads to large differences regarding elevated levels of FeNO in such a way that only 47 and 16% of subjects with FeNO above 25 ppb had levels above the 95th percentile in non‐sensitized and sensitized individuals, respectively. A FeNO value above the sensitisation‐specific 95th percentile was related to both having asthma and having current respiratory symptoms, but not to airways obstruction.

The effect of sensitisation on the upper limit defined as the 95th percentile of FeNO has not yet been extensively studied, especially in younger individuals. In adults, similar results with higher FeNO at the 95th percentile in individuals with atopy have been shown.^9^, ^17^, ^18^ As in the current study, sensitisation to perennial allergens, in particular that to furry pets, was associated with the highest FeNO median and 95th percentile values also in a cohort of an older Swedish population.^9^ In that study, respiratory healthy 50‐ to 64‐year‐olds that were sensitized to perennial allergens (analysis was limited to cat and mite) presented with higher FeNO, while this was not the case for those sensitized to seasonal allergens (birch), when compared to those without sensitisation. In another study of adults ages 29–54 years, Malinovschi et al.^19^ reported that out of cat, mite, mold, and timothy sensitisation, cat was the most closely related to FeNO. Further, in children and adults with asthma, Patelis et al.^20^ found that IgE sensitisation to mold, furry animals, and food allergens was associated with FeNO, while this was not seen for pollens. Results from the large European Community Health Respiratory Survey III study on healthy adults reported that those sensitized to both grass and perennial allergens had the highest FeNO levels.^21^ Hence, it seems reasonable to assume that in both adults and adolescents, the impact on FeNO of seasonal allergens is of less importance than to that of perennial such.

Given the close relationship between sensitization and FeNO, the odds ratios for respiratory symptoms and airway obstruction were calculated for sensitized and non‐sensitized groups separately in the current study. The estimates for asthma symptoms in the non‐sensitized group should be interpreted in the perspective that no more than 6–12% of the individuals in this group had a FeNO above the cutoff, set as either the 95th percentile or 25 ppb. The cutoff of 50 ppb did not allow for further analysis, as less than five individuals in this group presented with FeNO values above this level. Hence, the value of FeNO in the non‐sensitized subgroup is less clear than in the sensitized group, where individuals with FeNO over any of the cutoffs were between 2 and 4 times as likely to present with asthma or asthma symptoms, with prevalences up to 44% of those with a FeNO >50 ppb. The relationship between FeNO and asthma onset in general or non‐asthmatic populations is scarce; however, high FeNO has been associated with increased risk of new‐onset asthma^22^ and new‐onset wheeze.^23^ While sensitization was not assessed per se in those studies, the effect did not vary with history of respiratory allergic symptoms, supporting our results that FeNO may have a value in predicting asthma disease, and that this is not only mediated through sensitization status.

Finally, we found no increase in the probability of concurrent airway obstruction in those with FeNO values above either cut‐off, regardless of sensitization status. A relation between FeNO and lung function was not so consistently reported in the literature. For example, in subjects with asthma, no relation was found with airway obstruction at rest in a large group of asthmatic patients,^24^ while Sippel et al.^25^ reported a weak negative association, also in subjects with asthma. The lack of association in our study might be due to the fact that the majority of our participants had normal lung function. Another potential explanation is that some of the individuals with poor lung function do not have asthma, as it is acknowledged that poor lung function tracks with age and can be due to other factors such as genetics, early life risk exposures (smoking, air pollutants) and/or childhood respiratory infections (e.g., RSV or rhinovirus bronchiolitis).^26^, ^27^

### 4.1 Study limitations

A limitation in the current study may be that the measurements of FeNO were performed with a chemiluminescence analyzer, which is today likely to be less frequently used in clinical practice compared to analyzers using electrochemical sensors. However, it is likely that this is not affecting the findings of our study as an effect of IgE sensitization was found in a middle‐aged population when electrochemical sensors were used.^9^

The prevalences of different allergens are likely to vary between countries and continents.

Due to the cold weather, mites are relatively uncommon in Sweden where the study was taking place, as indicated by the low sensitisation rates for these allergens.^28^ Therefore, the results specific to mites need to be viewed in the perspective of the limited power. Further, the majority of the BAMSE participants are of European ancestry, and the results might not be generalizable beyond that. Finally, while the general population setup of the current study is beneficial in the estimation of reference limits, it may not be optimal for prediction of asthma disease or lung function impairment as power will be limited.

## 5 CONCLUSIONS

We have shown that the FeNO upper limit of normal is dependent on the presence and type of allergic sensitisation and that these cutoffs differ from the fixed cutoffs of 25 and 50 ppb recommended in the literature. Our results suggest that sensitisation specific, rather than overall fixed limit cutoffs, are more relevant regarding asthma diagnosis and asthma symptoms in a general population. Further studies are needed to establish cut‐offs in populations of a larger age range and to confirm the results of the present study.

## AUTHOR CONTRIBUTIONS

**Jenny Hallberg:** Conceptualization; methodology; data curation; investigation; formal analysis; writing – original draft; writing – review and editing; visualization. **Gang Wang:** Formal analysis; writing – review and editing. **Anna Bergström:** Methodology; funding acquisition; project administration; resources; writing – review and editing. **Inger Kull:** Methodology; funding acquisition; project administration; resources; writing – review and editing. **Erik Melén:** Methodology; funding acquisition; project administration; resources; writing – review and editing. **Andrei Malinovschi:** Supervision; formal analysis; conceptualization; methodology; visualization; writing – original draft; writing – review and editing.

## FUNDING INFORMATION

This study was supported by The Swedish Research Council, The Swedish Heart‐Lung Foundation, Region Stockholm (ALF project and database management), and the Swedish Asthma and Allergy Association. Thermo Fisher Scientific kindly provided the reagents for IgE analyses. None of the funding sources had a role in the study design, conduct, analysis, or reporting.

## CONFLICT OF INTEREST STATEMENT

EM has received lecture and/or advisory board fees from ALK, AstraZeneca, and Sanofi outside the submitted study. AM has received lecture and/or advisory board fees from Boehringer Ingelheim and Chiesi outside the submitted study as well as in‐kind support in the form of nitric oxide sensors from NIOX (a producer of FeNO devices) within the frame of an investigator‐initiated study.

## Supporting information


Appendix S1.
