# Supplementary material for: Importance of allergic sensitisation for normal range of fractional exhaled nitric oxide in adolescents
Source: Pediatr Allergy Immunol. 2025 Aug 1;36(8):e70154. doi: 10.1111/pai.70154 (PMC12314734; doi:10.1111/pai.70154)
Supplement: Supplementary file 1 — Appendix S1. [file PAI-36-e70154-s001.docx]

**Online material**

Importance of allergic sensitisation for normal range of fractional exhaled nitric oxide (FeNO) in adolescents

Jenny Hallberg, Gang Wang, Anna Bergström, Inger Kull, Erik Melén, Andrei Malinovschi

Table S1. Influence (beta-coefficient, 95% CI) of anthropometric variables on FeNO (ppb) in males and females in the reference population (n=1410).

|  | Females | | Males | |
| --- | --- | --- | --- | --- |
|  | 50^th^ percentile | 95^th^ percentile | 50^th^ percentile | 95^th^ percentile |
| Age (years) | -1.52 (-2.98;-0.05) | -8.87 (-26.22;8.62) | -0.07 (-1.85;1.72) | -6.73 (-22.18;8.72) |
| Height (cm) | 0.16 (0.07;0.26) | 0.14 (-0.96;1.25) | 0.18 (0.09;0.28) | 0.66 (-0.09;1.42) |
| Weight (kg) | 0.03 (-0.30;0.10) | -0.34 (-1.09;0.41) | 0.11 (0.04;0.17) | 0.49 (-0.00;0.98) |

Table S2. Influence of sensitization patterns on the 50^th^ and 95^th^ percentiles of FeNO (ppb) at 16 yrs in non-smoking females and males without asthma.

|  | n | FeNO 50^th^ perc.* | p value | FeNO 95^th^ perc. | p-value |
| --- | --- | --- | --- | --- | --- |
| **Females** |  |  |  |  |  |
| Phadiatop negative | 481 | 12.65 | Ref. | 26.85 | Ref. |
| Phadiatop positive | 241 | 14.85 | <0.001 | 54.65 | <0.001 |
| Perennial | 141 | 18.65 | <0.001 | 70.6 | <0.001 |
| Dog | 85 | 22.3 | <0.001 | 89.05 | <0.001 |
| Cat | 68 | 22.75 | <0.001 | 70.6 | <0.001 |
| Horse | 37 | 27 | <0.001 | 126.1 | <0.001 |
| House dust mite ^#^ | 59 | 17.1 | 0.001 | 74.5 | <0.001 |
| Mould^&^ | 3 | - |  | - |  |
| Seasonal | 172 | 16.88 | <0.001 | 56.95 | <0.001 |
| Examined in march-sep | 89 | 15.55 | 0.001 | 56.95 | <0.001 |
| Seasonal only | 76 | 12.88 | 0.232 | 27.7 | 0.827 |
| Examined in march-sep | 41 | 12.85 | 0.098 | 25.1 | 0.727 |
| **Males** |  |  |  |  |  |
| Neg. Phadiatop | 391 | 15.05 | Ref. | 33.45 | Ref. |
| Pos. Phadiatop | 297 | 19.9 | <0.001 | 62.45 | <0.001 |
| Perennial | 204 | 20.9 | <0.001 | 78.7 | <0.001 |
| Dog | 112 | 24.03 | <0.001 | 86.65 | <0.001 |
| Cat | 96 | 23.8 | <0.001 | 90.25 | <0.001 |
| Horse | 43 | 40.7 | <0.001 | 101.45 | <0.001 |
| House dust mite ^#^ | 96 | 19.325 | 0.013 | 84.60 | <0.001 |
| Mould^&^ | 10 | 39.38 | <0.001 | 101.45 | 0.026 |
| Seasonal | 217 | 20.15 | <0.001 | 60.6 | <0.001 |
| Examined in march-sep | 129 | 20.3 | <0.001 | 68.4 | <0.001 |
| Seasonal only | 83 | 19.2 | 0.001 | 38.55 | 0.272 |
| Examined in march-sep | 48 | 19.05 | 0.010 | 39.15 | 0.279 |

Phadiatop positive was defined as IgE levels against Phadiatop ≥0.35 Phadia Arbitrary Units/l. For individual allergens, sensitisation was defined if IgE levels agains the specific allergen ≥0.35 kU/l.

* The 50^th^ percentile was adjusted for body weight in males and body height and age in females.

^#^ House dust mite - *Dermatophagoides pteronyssinus*

^&^ Mould – *Cladosporium herbarum*

Table S3. Influence of IgE tertiles on the 50^th^ and 95^th^ percentiles of FeNO (ppb) at 16 yrs in non-smoking subjects without asthma.

|  | n | FeNO 50^th^ perc.* | p value | FeNO 95^th^ perc. | p-value |
| --- | --- | --- | --- | --- | --- |
| **All** |  |  |  |  |  |
| Phadiatop 1st tertile (<1.39 PAU /L) | 180 | 14.75 | 0.037 | 41.85 | <0.001 |
| Phadiatop 2nd tertile (1.39-13.3) | 179 | 18.25 | <0.001 | 52.20 | <0.001 |
| Phadiatop 3rd tertile (>13.3) | 179 | 23.20 | <0.001 | 81.55 | <0.001 |
| **Females** |  |  |  |  |  |
| Phadiatop 1st tertile (<0.85 PAU /L) | 80 | 13.00 | 0.914 | 30.90 | 0.347 |
| Phadiatop 2nd tertile (0.85-9.28) | 80 | 15.45 | <0.001 | 63.35 | 0.001 |
| Phadiatop 3rd tertile (>9.28) | 81 | 19.85 | <0.001 | 60.1 | <0.001 |
| **Males** |  |  |  |  |  |
| Phadiatop 1st tertile (<2.35 PAU/L) | 99 | 18.05 | 0.001 | 47.35 | 0.003 |
| Phadiatop 2nd tertile (2.35-16.7) | 99 | 19.6 | 0.001 | 58.35 | <0.001 |
| Phadiatop 3rd tertile (>16.7) | 99 | 24.1 | <0.001 | 85.2 | <0.001 |

* The 50^th^ percentile was adjusted for body weight and height, age and sex in males and females when assessed together, for body weight in males and body height and age in females when assessed separately.
